# Clinical validation of a tissue-agnostic genome-wide methylome enrichment assay to monitor response to pembrolizumab

**DOI:** 10.1038/s41698-026-01327-y

**Published:** 2026-02-13

**Authors:** Eric Y. Stutheit-Zhao, Yongqi Zhong, Collin A. Melton, Elizabeth D. Lightbody, Michael A. Hinterberg, Yarong Wang, Owen Hall, Eduardo V. Sosa, Jeremy B. Provance, Junjun Zhang, Abel Licon, Zhihui Amy Liu, Albiruni R. Abdul Razak, Anna Spreafico, Philippe L. Bedard, Aaron R. Hansen, Stephanie Lheureux, Pamela S. Ohashi, Alan Williams, Scott V. Bratman, Brian A. Allen, Jing Zhang, Daniel D. De Carvalho, Anne-Renee Hartman, Lillian L. Siu, Enrique Sanz-Garcia

**Affiliations:** 1 Princess Margaret Cancer Centre, University Health Network, Toronto, ON Canada; 2 Adela, Inc., Foster City, CA USA; 3 Department of Immunology, University of Toronto, Toronto, ON Canada; 4 Department of Medical Biophysics, Temerty Faculty of Medicine, University of Toronto, Toronto, ON Canada

**Keywords:** Biomarkers, Cancer, Oncology

## Abstract

Immunotherapy has significantly improved the treatment of metastatic solid tumors; however, detecting early signs of response to enable timely intervention for resistant tumors remains challenging. A blood-only circulating tumor DNA (ctDNA) test may provide a rapid assessment of tumor response without reliance on matched tumor tissue. We applied a tissue-agnostic, genome-wide methylation enrichment assay, based on cell-free methylated DNA immunoprecipitation and high-throughput sequencing (cfMeDIP-seq), to plasma samples from patients in a phase 2 trial evaluating pembrolizumab across multiple solid tumors (NCT02644369). A decrease in ctDNA from baseline to pre-cycle 3 was significantly associated with higher objective response and clinical benefit rates and longer progression-free and overall survival in univariate analyses, with these associations remaining significant in multivariable models except for overall survival. These results validate a commercial-grade, tissue-agnostic plasma cfDNA methylation platform for immunotherapy response monitoring, which may facilitate earlier, more informed treatment decisions and improve patient outcomes.

## Introduction

Immune checkpoint inhibitors (ICIs), including programmed cell death protein 1 and programmed death-ligand 1 (PD-1/PD-L1) inhibitors, have produced durable responses and improved outcomes for patients with various cancer types^1–3^. Despite major advancements, heterogeneity in clinical responses remains a challenge^4^. Because only a subset of patients will benefit from ICI therapy, biomarkers capable of early identification of treatment efficacy may provide opportunities to maximize clinical benefit. Currently, there are few established predictive biomarkers for ICI response, including tumor PD-L1 expression and tumor mutational burden (TMB)^5^. However, these biomarkers rely on tissue biopsies that are not always available, have not consistently predicted treatment response across all cancer types, and are mainly used as static baseline biomarkers rather than for dynamic response monitoring. Radiographic response assessment also has limitations, as scans can be confounded by immunotherapy-induced immune cell infiltration, potentially leading to inaccurate tumor size measurements and the appearance of pseudoprogression^6–8^. Moreover, imaging cannot be widely conducted to provide a rapid and repeated readout of disease burden. Therefore, there is an unmet need for reliable minimally invasive biomarkers capable of identifying primary resistance and disease progression early in the treatment paradigm to help guide treatment continuation or adaptation and improve the outcomes of a wider spectrum of patients.

Evaluation of circulating tumor DNA (ctDNA) dynamics is a useful approach for accurate real-time detection of tumor burden and assessment of clinical response to immunotherapeutic agents, especially in the advanced disease setting^9,10^. However, most widely used methods for quantifying ctDNA utilize a tumor-informed approach, where prior whole exome or genome sequencing (WES or WGS) of tumor biopsy tissue is required to identify patient-specific mutations and design personalized single-nucleotide variant (SNV) and structural variant (SV) panels for subsequent peripheral blood plasma profiling and ctDNA quantification^11,12^. Tumor-informed approaches may be impractical in many cases due to challenges in accessing tumor tissue, particularly in cancers where biopsies are anatomically difficult, infrequently performed, or yield insufficient material. Even when tissue is obtained, the quantity may not be adequate for both standard diagnostic workup and the additional genomic profiling needed for a tumor-informed strategy. Furthermore, the time required to perform upfront tumor WES or WGS and downstream bespoke panel design may pose logistical issues for response monitoring, where decisions may need to be implemented rapidly after only a few weeks and prior to radiographic assessments^13^. Thus, sensitive tissue-agnostic approaches for ctDNA assessment may be advantageous for routine use in clinical practice.

DNA methylation plays a critical role in the normal development and regulation of many cellular processes. Cancer-specific DNA methylation aberrations are stable, as well as cell-type and tissue-specific^14,15^. Cell-free DNA (cfDNA) methylation profiling can detect the presence of ctDNA for disease detection and monitoring with high sensitivity, eliminating the need for a reference tumor tissue biopsy^16–20^. One method for profiling the cfDNA methylome is cell-free methylated DNA immunoprecipitation followed by high-throughput sequencing (cfMeDIP-seq). This technique employs a tissue-agnostic, genome-wide methylation enrichment platform. Compared to other cfDNA methylation profiling approaches, cfMeDIP-seq offers several advantages: it avoids chemical or enzymatic conversion, thereby minimizing DNA degradation; it retains more ctDNA signal and fragment length information; enhances mapping efficiency; and selectively sequences only the methylated cfDNA fragments^19–21^. We and others have shown that this tissue-agnostic, genome-wide methylome enrichment platform has clinical utility across multiple cancer types and applications, including in a blinded study validating its performance for molecular residual disease (MRD) detection in head and neck cancer^19–28^. In addition to its potential for MRD, we recently demonstrated in the INSPIRE study (NCT02644369), at proof-of-concept level, that serial cfDNA methylation profiling with cfMeDIP-seq can also track immunotherapy responses in solid tumors^25^. However, this study utilized an academic cfMeDIP-seq protocol, which lacks the technical refinements necessary to be used in a clinical setting, along with a classifier limited to array-derived methylation features.

In this study, we applied a clinical-grade cfDNA methylation assay and classifier, which directly leverages cfMeDIP-seq data, to conduct a blinded, pre-defined analysis of samples from the investigator-initiated phase 2 INSPIRE study (NCT02644369)^29^. We profiled 241 plasma cfDNA methylomes at baseline and during single-agent treatment with pembrolizumab in 69 patients with diverse solid cancers, including patients with head and neck squamous cell carcinomas (HNSCC), triple-negative breast cancer (TNBC), high-grade serous ovarian cancer (HGSOC), melanoma, and mixed solid tumors (MSTs). This work clinically validates the use of a tissue-agnostic, genome-wide methylome enrichment platform to determine molecular treatment response using dynamic ctDNA monitoring in patients across various advanced solid cancer types treated with pembrolizumab.

## Results

### Characteristics of study cohorts with advanced solid tumors

**Fig. 1:**
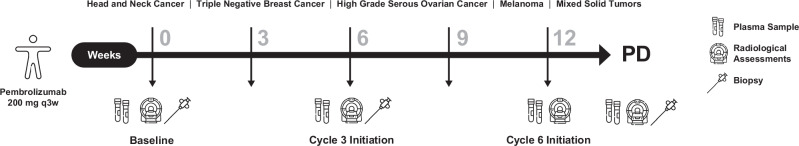
Study design. The INSPIRE study includes patients with advanced solid tumors, not previously treated with immunotherapy, who received pembrolizumab at a dosage of 200 mg every 3 weeks (q3w). PD progressive disease.

**Fig. 2:**
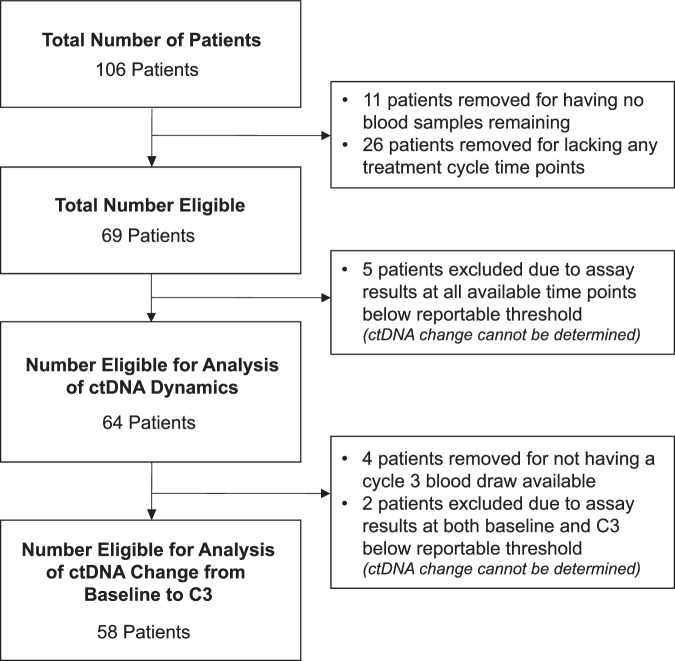
A CONSORT diagram showing the number of patients in the study. Patients and their associated banked samples are shown. C3 cycle 3, ctDNA circulating tumor DNA.

We conducted a blinded validation analysis of cfMeDIP-seq technology using Adela’s proprietary platform to quantify ctDNA levels in patients with advanced solid tumors enrolled in INSPIRE (NCT02644369), a single-institution, investigator-initiated phase 2 study evaluating immunological response to pembrolizumab. Patients provided informed consent and were enrolled in five parallel cohorts consisting of HNSCC (cohort A), TNBC (cohort B), HGSOC (cohort C), malignant melanoma (cohort D), and MSTs (cohort E). Blood samples were collected at baseline (prior to treatment initiation), every 3 cycles starting at cycle 3 (during treatment), and at disease progression (Fig. 1). Among 106 patients enrolled in INSPIRE, 69 had baseline and at least one post-baseline blood sample remaining, 64 patients had available results for analysis of ctDNA dynamics after excluding 5 patients due to assay results being below the reportable threshold at all available timepoints. Among the 64 patients, 58 had available results for analysis of the change in ctDNA scores from baseline to the cycle 3 timepoint, after excluding 6 patients due to no available cycle 3 blood sample or assay results below the reportable threshold at both timepoints (Fig. 2).

We compared baseline demographic, clinical, and outcome variables between the 58 patients included in the primary analyses and the 37 excluded patients with available clinical data. No statistically significant differences were observed across these characteristics, indicating that excluded patients did not differ meaningfully in baseline prognosis (Supplementary Table 1). These findings suggest that the exclusions did not introduce systematic bias into the analytic cohort.

Among the 64 patients eligible for analysis of ctDNA dynamics, 23.4% of patients (15/64) had an objective response (complete response, CR, or partial response, PR, at the best response cycle, Table 1), and 31.25% (20/64) had clinical benefit (objective response or stable disease, SD, lasting for ≥6 cycles). The follow-up time of patients eligible for ctDNA dynamic analysis ranged from 3.15 to 101.22 months with a median of 18.43 months, and the median follow-up time of alive patients eligible for analysis was 93.82 months as of December 31st, 2024. Median progression-free survival (PFS) and median overall survival (OS) were 1.97 months (95% CI: 1.94–4.17 months, range: 1.41–98.89 months) and 18.69 months (95% CI: 13.90–25.33 months, range: 3.15–101.22 months), respectively.

**Table 1:** Patient demographics and clinical characteristics

Characteristic	Patients *n* = 64^a^
Age, median	60.75
Sex, *n* (%)	
Female	35 (54.69%)
Male	29 (45.31%)
Cohort, *n* (%)	
A. Head and neck	11 (17.19%)
HPV+	4 (36.36%)
HPV-	1 (9.09%)
Unknown	4 (36.36%)
Missing	2 (18.18%)
B. Breast	10 (15.63%)
C. Ovarian	10 (15.63%)
D. Melanoma	8 (12.50%)
E. Mixed solid tumors	25 (39.06%)
Best overall response, *n* (%)	
Complete response	2 (3.13%)
Partial response	13 (20.31%)
Stable disease	16 (25.00%)
Progressive disease	33 (51.56%)
Biomarkers	
PD-L1 expression, mean % (SD)	16.16% (32.40%)
PD-L1 subgroups, *n* (%)	
<1%	33 (51.56%)
1–49%	22 (34.38%)
≥50%	9 (14.06%)
Tumor mutational burden, mean (SD)^b^	13.61 (52.58)

^a^Median follow-up among surviving patients, 93.82 months.

^b^*n* = 62 due to 2 patients not having tumor mutational burden data available.

### A decrease in ctDNA scores from baseline to cycle 3 is associated with better treatment response and prognosis

**Fig. 3:**
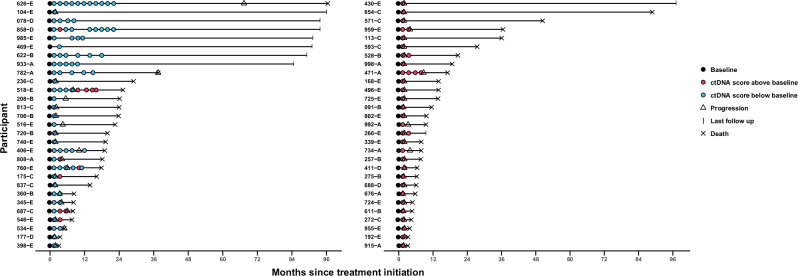
Relative ctDNA scores in serial blood draws over the course of treatment. Swimmer plots depicting on-treatment ctDNA score relative to the baseline ctDNA score in available serial blood draws over the course of treatment in each patient eligible for analysis of ctDNA change from baseline to C3 (*n* = 183 samples from 58 patients). Each row represents a unique patient participant (y-axis) where patients are grouped by ctDNA score status at cycle 3 and ordered by total length of follow-up since treatment initiation (x-axis, months). Patients with a ctDNA score <baseline ctDNA score at cycle 3 (*n* = 29) (blue circles; left panel) and patients with a ctDNA score >baseline ctDNA score at cycle 3 (*n* = 29) (pink circles; right panel) are shown. Black circles indicate baseline ctDNA samples, triangles indicate disease progression, ‘X’ indicates death events, and tick marks represent last follow-up.

Patients were categorized into two groups based on the numerical change in ctDNA scores from baseline to cycle 3: increased (absolute cycle 3 ctDNA score - absolute baseline ctDNA score >0) and decreased (absolute cycle 3 ctDNA score - absolute baseline ctDNA score <0). We found that 50.00% of the patients (*n* = 29/58) showed a decrease in ctDNA from baseline to cycle 3, while the other 50.00% showed an increase (Fig. 3**;** Supplementary Fig. 1). When stratified by cancer type, no statistically significant differences were observed among the five pembrolizumab-treated cohorts in the proportion of patients demonstrating ctDNA decrease versus increase from baseline to cycle 3 **(**Supplementary Table 2**)**. Similarly, the magnitude of ctDNA change, assessed using both absolute change and fold change, did not differ significantly across cancer types **(**Supplementary Fig. 2**)**.

**Fig. 4:**
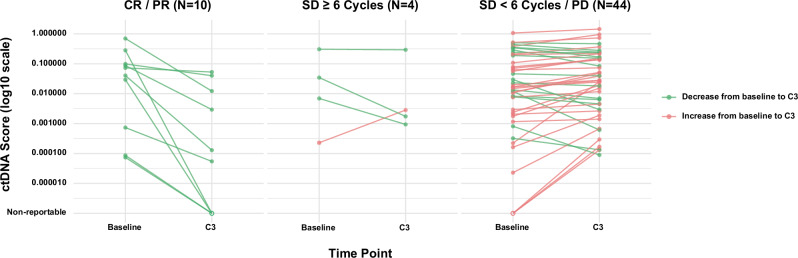
Trajectory of baseline and cycle 3 ctDNA scores by response status. ctDNA dynamics between baseline and C3 timepoints in patients with available results (*n* = 58). ctDNA scores are represented in log10 scale (y-axis) between timepoints (x-axis) and stratified according to treatment response groups. Left panel: Complete Response/Partial Response; Middle panel: Stable Disease ≥6 cycles; Right panel: Stable Disease <6 cycles/Progressive Disease. ctDNA scores decreased from baseline to C3 are presented in green lines, whereas those that increased are in red. Hollow dots represent ctDNA scores below the reportable threshold, while solid dots are ctDNA scores above. C3 cycle 3, CR complete response, PD progressive disease, PR partial response, SD stable disease.

Among the 29 patients with an increase in ctDNA scores, none achieved an objective response, and 96.55% (28/29) did not experience clinical benefit (Fig. 4). In contrast, all patients (10/10) with an objective response and 92.86% (13/14) of those who experienced clinical benefit had a decrease in ctDNA. Firth’s penalized logistic regressions showed that patients with a decrease in ctDNA scores from baseline to cycle 3 were more likely to achieve an objective response (odds ratio [OR] = 31.77, 95% CI: 3.71–4173.19, *P* = 0.0003) and clinical benefit (OR = 15.55, 95% CI: 3.31–151.52, *P* = 0.0002), compared to those with an increase in ctDNA. Similar associations were observed in objective response (adjusted OR = 33.51, 95% CI: 3.83–4407.64, *P* = 0.0003) and clinical benefit (adjusted OR = 18.89, 95% CI: 3.64–222.98, *P* = 0.0001) when adjusting for cohort (Supplementary Tables 3 and 4).

**Fig. 5:**
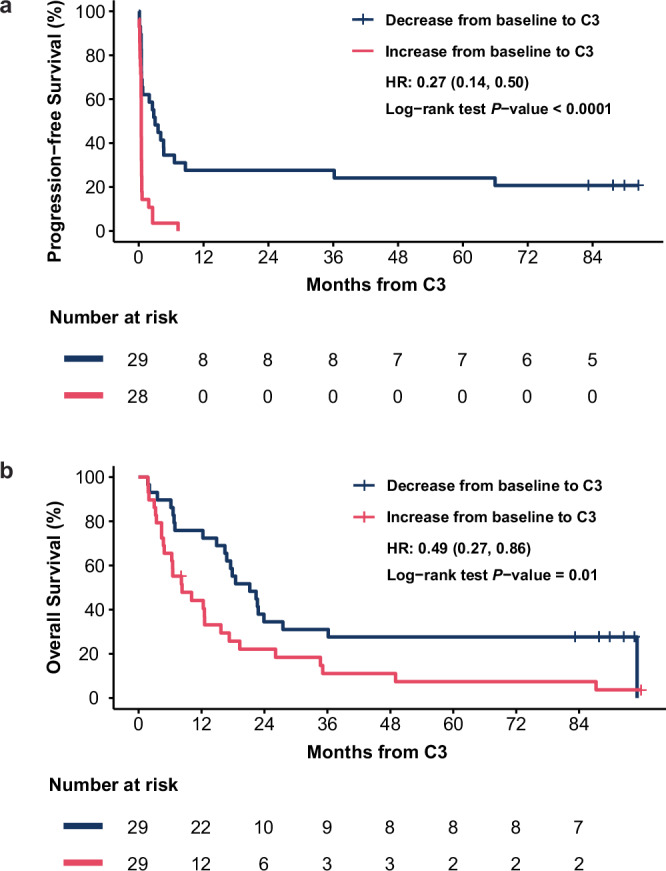
ctDNA dynamics between baseline and C3 are strongly correlated with PFS and OS. **a** Kaplan–Meier curve of PFS by change in ctDNA score from baseline to C3 (increase vs. decrease; *n* = 57) and **b** OS by change in ctDNA score from baseline to C3 (increase vs. decrease; *n* = 58). Increase in ctDNA from baseline to C3 is represented in pink, and decrease is represented in blue. One patient was excluded from the PFS analysis due to progression before C3. Statistical significance was evaluated using a two-sided log-rank test. HRs for PFS and OS were estimated using Cox proportional hazard models. C3 cycle 3, HR hazard ratio, OS overall survival, PFS progression-free survival.

Furthermore, Cox proportional hazard models and log-rank tests indicated that a decrease in ctDNA scores was associated with improved PFS (hazard ratio [HR] = 0.27, 95% CI: 0.14–0.50, *P* < 0.0001) and OS (HR = 0.49, 95% CI: 0.27–0.86, *P* = 0.01) (Fig. 5). Similar associations were found in the cohort-adjusted PFS (adjusted HR = 0.21, 95% CI: 0.11–0.43, *P* < 0.0001) and OS (adjusted HR = 0.45, 95% CI: 0.25–0.83, *P* = 0.01) (Supplementary Tables 5 and 6).

### A decrease in ctDNA scores from baseline to cycle 3 is strongly associated with clinical outcomes, alongside other immune checkpoint blockade biomarkers

We further investigated treatment response and clinical outcomes by evaluating early ctDNA dynamics alongside other relevant variables, such as cohort, PD-L1 expression, and TMB. In univariate analyses (Supplementary Tables 3–6), we found that, in addition to the change in ctDNA scores from baseline to cycle 3, higher PD-L1 expression was significantly associated with objective response (OR = 1.03, 95% CI: 1.01–1.05, *P* = 0.004), clinical benefit (OR = 1.03, 95% CI: 1.01–1.05, *P* = 0.002), and favorable PFS (HR = 0.98, 95% CI: 0.97–1.00, *P* = 0.01).

Multivariable analyses were conducted to adjust for covariates (Supplementary Tables 3–6) and to compare regression models with and without the inclusion of changes in ctDNA scores from baseline to cycle 3. After adjustment for covariates, a decrease in ctDNA scores remained strongly and independently associated with objective response (*P* = 0.01), clinical benefit (*P* = 0.004), and PFS (*P* = 0.0006), but showed no significant association with OS (*P* = 0.17). Likelihood ratio tests indicated that regression models incorporating changes in ctDNA scores significantly improved model fit for associations with objective response (*P* = 0.0009), clinical benefit (*P* = 0.002), and PFS (*P* = 0.0004), but not for OS (*P* = 0.17). These results demonstrate a highly significant improvement in the associations with treatment response and outcomes when ctDNA score changes are included.

### Low ctDNA scores consistently below baseline across all treatment cycles are prognostic for improved PFS and OS

**Fig. 6:**
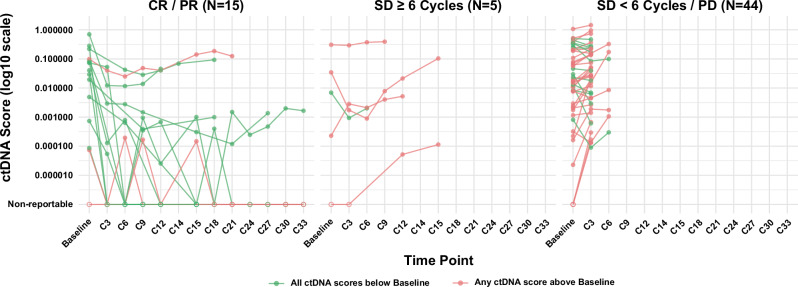
Trajectory of ctDNA dynamics over the course of treatment by response status. ctDNA dynamics between baseline and post-baseline timepoints in all analyzable patients (*n* = 64). ctDNA scores are represented in log10 scale (y-axis) between timepoints (x-axis) and grouped by treatment responses. Left panel: Complete Response/Partial Response; Middle panel: Stable Disease ≥6 cycles; Right panel: Stable Disease <6 cycles/Progressive Disease. ctDNA scores that are consistently below baseline are presented in green lines, whereas those with any ctDNA scores above baseline are in red. Hollow dots represent ctDNA scores below the reportable threshold, while solid dots are ctDNA scores above. Patients with baseline ctDNA score below the threshold and any post-baseline score above the threshold were included because values exceeding the threshold indicate evidence of disease, allowing for qualitative assessment of the change (i.e., increase from baseline). CR complete response, EOT end of treatment, PD progressive disease, PR partial response, SD stable disease.

**Fig. 7:**
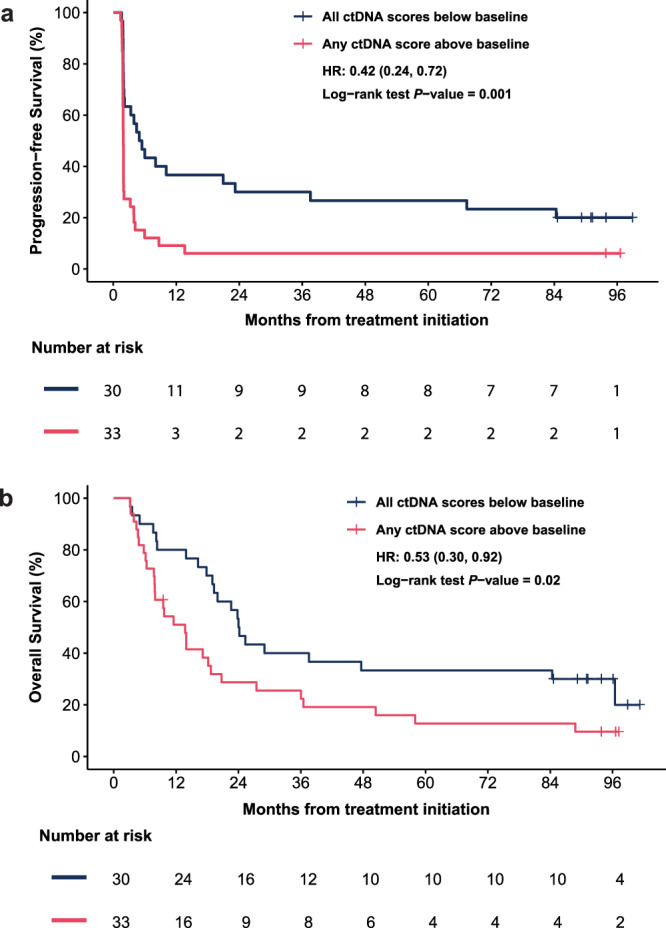
Kaplan–Meier estimates of time to event by serial ctDNA dynamics relative to baseline. **a** PFS stratified by post-baseline ctDNA scores relative to baseline prior to progression, death, or the end of FU, whichever occurred first (*n* = 63). **b** OS stratified by post-baseline ctDNA scores relative to baseline (*n* = 63). Serial ctDNA status was categorized as either all blood draws show a decrease after the baseline timepoint (blue) or any blood draw shows an increase after the baseline timepoint (pink). The statistical significance of the difference between the two curves was assessed using a two-sided log-rank test. HRs for PFS and OS were estimated using Cox proportional hazards models. HR hazard ratio, OS overall survival, PFS progression-free survival, FU follow-up.

To provide a comprehensive profile of ctDNA dynamics over the course of pembrolizumab treatment, we visualized the ctDNA scores for each patient with available results (*n* = 64) (Fig. 6). Although ctDNA scores fluctuated throughout the course of treatment, 46.88% of patients eligible for all timepoint ctDNA dynamic analysis (*n* = 30/64) had post-baseline ctDNA scores that remained consistently below their baseline values before progression, death, or the end of follow-up, whichever occurred first. In contrast, 51.56% of patients eligible for all timepoint ctDNA dynamic analysis (*n* = 33/64) had ≥1 ctDNA score exceeding their baseline level before progression. Only one patient experienced progression prior to the cycle 3 blood draw (i.e., the earliest post-baseline blood draw). Of note, 80.00% (12/15) of patients with an objective response exhibited post-baseline ctDNA scores consistently below their baseline values. In contrast, 70.45% (31/44) of patients with SD <6 cycles or progressive disease had at least one post-baseline ctDNA score above baseline. As expected, patients with consistently below-baseline ctDNA scores prior to progression exhibited significantly improved PFS (HR = 0.42, 95% CI: 0.24–0.72, *P* = 0.001) and OS (HR = 0.53, 95% CI: 0.30–0.92, *P* = 0.02), compared to those with any ctDNA scores above baseline (Fig. 7). These associations provide important analytical and clinical confirmation that the assay captures meaningful changes in tumor burden over time in addition to important early ctDNA decreases.

Next, we evaluated patients with ctDNA results below the reportable threshold through both descriptive and comparative analyses. In a descriptive review of the seven patients with ctDNA below the reportable threshold at baseline and/or other timepoints, patients whose ctDNA remained undetectable across timepoints generally experienced clinical benefit, whereas those with a subsequent increase in ctDNA from baseline were more likely to develop progressive disease and have poorer survival outcomes. Thus, below-threshold ctDNA values in this cohort were not uniformly attributable to an indolent or “non-shedder” phenotype, as both aggressive and favorable trajectories were observed (Supplementary Table 7**)**. Meanwhile, a comparative analysis demonstrated that patients with ctDNA below the reportable threshold (*n* = 7) had significantly higher rates of clinical benefit (CR/PR/SD ≥6 cycles) and significantly lower rates of disease progression compared with patients included in the primary analysis (*n* = 58), along with non-statistically significant trends toward higher objective response rates and fewer death events (Supplementary Table 8). Although this analysis is exploratory and limited by the small sample size, it suggests a potential association between undetectable ctDNA at baseline and improved clinical outcomes.

## Discussion

We performed a blinded validation analysis to demonstrate the clinical utility of measuring ctDNA dynamics for immunotherapy response monitoring in a cohort of patients with various solid cancer types. This study further advances our prior analyses in the INSPIRE cohort by establishing a clinical-grade cfDNA methylation assay with improved quality and reproducibility, replacing earlier research-grade cfMeDIP-seq^25^. Additionally, instead of relying on a classifier trained using fragment length features and tissue-derived methylation arrays that capture only a small fraction of the human methylome, we developed a proprietary algorithm powered by our extensive plasma cfMeDIP-seq dataset^21,30,31^. By leveraging cfMeDIP data directly, rather than relying on orthogonal data types and fixed differentially methylated region (DMR) arrays, we enable more robust identification of high-signal and low-noise DMRs in cfDNA. Our overall approach enables fully blood-based, tissue-agnostic methylome profiling with direct translational potential for early assessment of ICI treatment response.

In this study, a decrease in ctDNA score from baseline to cycle 3 was associated with improved treatment response, as patients with a decrease were more likely to achieve both an objective radiological response and clinical benefit from pembrolizumab. Meanwhile, among all patients with an increase in ctDNA between baseline and cycle 3, 100.00% of patients did not achieve an objective response, and 96.55% did not derive clinical benefit. This study also demonstrated that a decrease in ctDNA from baseline to cycle 3 was associated with improved PFS and OS in univariate analyses. After adjusting for established clinical variables relevant to immunotherapy response, including TMB and PD-L1 expression, the multivariable model indicated that a decrease in ctDNA from baseline to cycle 3 contributed independently to the association with objective response, clinical benefit, and PFS. In fact, an early ctDNA decrease demonstrated the strongest and most consistent association with clinical outcomes, with the exception of OS. The extended follow-up period may explain why an early ctDNA decrease was not associated with OS in the multivariable analysis, as most patients eventually experienced disease progression, making post-progression events and subsequent treatments the major drivers of survival outcomes. Thus, the loss of significance for OS in the multivariable model is likely attributable to these confounding influences or to limited power, rather than an absence of underlying signal. Overall, these findings are consistent with prior studies demonstrating that early decreases in ctDNA are associated with improved outcomes in patients treated with immunotherapy, reinforcing this well-established biological relationship^32,33^.

In contrast, the lack of statistical significance for TMB and PD-L1 in multivariable analyses of objective response, clinical benefit, or PFS suggests that these biomarkers do not contribute independently to these outcomes when accounting for other covariates. These results align with prior findings that TMB and PD-L1 expression in tissue or blood are informative in only a subset of tumors and have not served as robust, real-time indicators to guide treatment decisions after therapy initiation^34–36^. Notably, the limited number of response events resulted in an events-per-variable (EPV) ratio below recommended thresholds, and the multivariable models may therefore be over-parameterized. Although we used Firth’s penalized regression to reduce small-sample bias, it cannot eliminate the underlying instability reflected in the wide confidence intervals, and point estimates should therefore be interpreted with caution^37^. Nonetheless, sensitivity analyses with alternative covariate adjustments produced consistent directional associations across endpoints, supporting the robustness of our findings.

Next, we conducted a descriptive analysis of ctDNA dynamics over the course of pembrolizumab treatment. Patients who showed a complete or partial clinical response generally showed large decreases or moderate increases in serial ctDNA levels compared to their baseline ctDNA level. In contrast, patients with stable or progressive disease showed smaller decreases or larger increases in ctDNA levels compared to their baseline level. Patients with consistently below-baseline ctDNA levels exhibited significantly improved PFS and OS. These results suggest that ctDNA dynamics may be associated with progression and survival outcomes and help identify subsets of patients who are likely to benefit from ICI treatment. Clinical utility should be confirmed through validation in prospective interventional studies.

Our findings are consistent with a previous analysis of a patient subset from the same INSPIRE study, which used a tumor-informed ctDNA profiling approach based on patient-specific variants and reported that ctDNA dynamics were associated with treatment response and clinical outcomes with pembrolizumab^32^. In that tissue-informed analysis, a decrease in ctDNA from baseline to cycle 3 was associated with improved OS (HR 0.36, 95% CI 0.18–0.71) and PFS (HR 0.33, 95% CI 0.19–0.58). In our study, a decrease in ctDNA from baseline to cycle 3 was likewise significantly associated with favorable OS (HR 0.49, 95% CI 0.27–0.86) and PFS (HR 0.27, 95% CI 0.14–0.50). Notably, while these studies included a significant overlap in patients, differences in sample availability and assay requirements resulted in non-identical cohorts. Nonetheless, the approach used in our study demonstrated effect sizes and prognostic performance comparable to a tissue-informed method, despite concerns about detection limits for tumor-agnostic ctDNA assays, while also introducing clinically meaningful advantages for response monitoring. First, this assay can be applied to a broader range of patients, including those without adequate tumor tissue, thereby increasing accessibility and expanding eligibility for ctDNA-based monitoring. Second, its consistent performance across a heterogeneous cohort of cancer types provides evidence for the broad applicability of this approach across tumor types. Lastly, tumor-agnostic approaches generally have a shorter turnaround time compared to tissue-informed approaches since they bypass the need for upfront tumor sequencing for patient-specific panel design, which may enable more rapid deployment in clinical workflows^38^. This may be particularly advantageous for response monitoring, where early changes, such as those observed from baseline to cycle 3 in our study, may be clinically informative.

This study has many strengths, including standardized frequent sampling timepoints, uniform single-agent administration, inclusion of multiple tumor types, an assay with low cfDNA input requirements, and extended follow-up time that enabled significant clinical outcome analysis. Additionally, immune-driven cfDNA resulting from immunotherapy-induced immune cell expansion is unlikely to contribute to the observed ctDNA signals, given that classifier DMRs were explicitly filtered to exhibit minimal methylation in peripheral blood leukocytes. Limitations of the study include incomplete sample availability from the full cohort of 106 enrolled patients and the absence of earlier on-treatment timepoints prior to cycle 3, which may have provided additional value for earlier clinical decision-making. Although this study included a heterogeneous population of advanced solid tumors, the absence of a dedicated NSCLC cohort is a meaningful limitation, particularly given that NSCLC represents one of the largest patient populations treated with pembrolizumab^39–42^. As a result, the findings reported here should not be generalized to pembrolizumab-treated lung cancer without dedicated validation in NSCLC. In the future, larger multi-center prospective studies that include additional cancer types across different treatment indications and heterogeneous molecular subtypes will be required for broader applicability, to strengthen the observed associations reported in this study, and to allow for adequately powered subgroup analyses. Since the ctDNA analysis from INSPIRE was conducted retrospectively, it remains undetermined how our ctDNA-informed response monitoring could affect treatment decision-making. Rationally designed interventional trials are necessary to progress ctDNA-based monitoring assays for risk-stratified decision-making across cancers.

In summary, these results validate the clinical performance of a tissue-agnostic genome-wide methylome enrichment commercial-grade assay for immunotherapy response monitoring. This assay confirms that dynamic ctDNA levels and early reductions in ctDNA following treatment with single-agent pembrolizumab are associated with longer PFS and OS across multiple cancer types. Overall, the approach may enable clinicians to make earlier treatment decisions, optimize therapy selection, and improve patient outcomes through accessible molecular disease monitoring.

## Methods

### Study design

This study utilized samples and clinical data from the INSPIRE study, a single-institution investigator-initiated phase 2 study of pembrolizumab immunological response evaluation in multiple solid tumors (NCT02644369). Participants provided informed consent to participate in the study, which was approved by the Research Ethics Board at the Princess Margaret Cancer Centre and was conducted in accordance with the principles of Good Clinical Practice, the provisions of the Declaration of Helsinki and its amendments, and other applicable local regulations.

### Genome-wide methylome enrichment platform

All ctDNA samples were analyzed with a bisulfite-free, non-degradative genome-wide DNA methylation enrichment platform based on cfMeDIP-seq, using 5–10 ng of cfDNA extracted from plasma as previously described^19–21^. The test described in this clinical validation study is not yet commercially available, and assessments of analytical performance are ongoing. However, the clinical performance of this assay in the INSPIRE cohort includes a sensitivity of 0.60 (29/48) (95% CI, 0.45–0.74) for detecting progression or stable disease in patients with no objective response; specificity was 1.00 (10/10) (95% CI, 0.66–1.00) for the detection of objective response. This assay is expected to have a turnaround time of approximately 7 business days based on experience with similar tests and preliminary results from this study.

### Classifier development and ctDNA quantification

Prior to unblinding data for clinical validation analysis, independent training cohorts were used to train a classifier to measure ctDNA abundance in cfDNA^21,30,31^. Using cfMeDIP-seq, cancer-specific DMRs were identified in samples from individuals with cancer and non-cancer, including pre-treatment, post-treatment, recurrent, and post-treatment non-recurrent samples. DMRs were selected to exhibit low background signal in cfDNA from non-cancer individuals. Specificity was confirmed via an exploratory analysis mapping classifier DMRs to CpG sites on Illumina 450K arrays, demonstrating tumor specificity of methylation signals vs peripheral blood leukocytes or normal tissues. In the current study, the ctDNA score for a given sample of interest was determined following cfMeDIP-seq by normalizing the number of sequenced fragments mapping to cancer-specific DMRs to other control regions and to the total number of uniquely sequenced molecules. The lower threshold for reporting of scores was set at the point where the classifier is effectively calling a zero-tumor fraction. A reportable threshold with a numeric score value of 0.00001 was chosen to avoid reporting results for samples where the observed number of fragments in DMRs is equal to or less than the expected number of fragments in a blank/non-cancer sample. The classifier represents a product-grade implementation, supported by analytically validated assay protocols, quality-controlled pipelines, and reproducible performance across multiple batches and operators.

### Study objectives and clinical endpoints

The co-primary objectives for this study were to determine whether a decrease in ctDNA scores by cycle 3 from baseline, defined as absolute ctDNA score at cycle 3-absolute ctDNA score at baseline <0, was significantly associated with a difference in objective response and PFS through response monitoring. Objective response was defined as a patient who experienced CR or PR at the best response cycle as determined by radiological assessment. PFS was defined as the time from the first infusion to a progression event, death, or censoring time, whichever occurred first. Progression event was defined as disease progression based on RECIST (v1.1), or clinical progression if the patient discontinued pembrolizumab due to clinical deterioration despite not meeting criteria for progression. The secondary objectives for this study were to determine whether a decrease in ctDNA levels by cycle 3 from baseline was significantly associated with better clinical benefit or better OS. Clinical benefit was defined as a patient who experiences CR, PR, or SD lasting for ≥6 cycles, whereas OS was defined as the time from the first infusion to death or last follow-up, whichever occurred first. Lastly, we aimed to determine whether any increase in ctDNA levels during any treatment cycle compared to baseline was significantly associated with worse PFS and OS. Patients with all ctDNA scores below the reportable threshold were excluded, as a change in ctDNA could not be determined. Patients with one ctDNA score below the threshold and one above the threshold were included, allowing for qualitative assessment of the change.

Trajectories of ctDNA levels by treatment response status over the course of treatment and associations of ctDNA level, as well as clinical outcomes (objective response, clinical benefit, PFS, OS), adjusting for cohort, PD-L1, and TMB, were exploratory endpoints. TMB was assessed via WES. PD-L1 expression was analyzed using PD-L1 immunohistochemistry (clone 22C3) and quantified using the modified proportion score (MPS) calculated by Qualtek (Goleta, CA). MPS is based on the proportion of PD-L1-expressing tumor cells and mononuclear inflammatory cells found within tumor nests, as previously described^29^.

### Statistical methods

In a blinded validation analysis, a prespecified statistical analysis plan was finalized and approved prior to data analysis. Fixed sequence testing was employed to control the family-wise error rate at 0.05 for the 2 prespecified co-primary analyses evaluating changes in ctDNA scores from baseline to cycle 3, with respect to objective response and PFS. The analysis for PFS was performed only if the analysis for objective response reached statistical significance. Power calculations were conducted to ensure ≥80% probability of detecting a statistically significant difference in both objective response and PFS. Descriptive statistics were used to summarize demographic and baseline characteristics. The median PFS and the median OS were estimated because more than half of the patients experienced progression or death.

Firth’s logistic regression was employed to assess differences in objective response and clinical benefit between patients who experienced a decrease in ctDNA score from baseline to cycle 3 and those who had an increase. This method was selected to reduce bias in estimating ORs in the presence of small sample sizes or complete separation (i.e., low or zero cell counts in the 2 × 2 contingency table), thereby improving the robustness of the estimates^37,43^. Although we used Firth’s penalized regression, it cannot eliminate the underlying instability reflected in the wide confidence intervals, and point estimates should therefore be interpreted with caution. Statistical significance was evaluated using a two-sided penalized likelihood ratio test with an alpha level of 0.05.

The Kaplan–Meier method was used to estimate and compare PFS (from the start of cycle 3) and OS (from the start of cycle 3) between patients whose ctDNA scores increased at cycle 3 and those whose scores decreased. Statistical differences were tested using the log-rank test at a two-sided alpha level of 0.05. Cox proportional hazards models were employed to estimate unadjusted and cohort-adjusted HRs for PFS (from the start of cycle 3) and OS (from the start of cycle 3). Additionally, PFS (from the first infusion) and OS (from the first infusion) were compared between patients whose ctDNA scores were larger than the baseline value at any point during treatment versus ctDNA scores that were always below baseline before the corresponding clinical event (e.g., progression, death).

We conducted multivariable analysis on objective response, clinical benefit, PFS, and OS, adjusting for cohort, PD-L1 expression, and TMB. A (penalized) likelihood ratio test comparing nested models was conducted to determine whether incorporating early ctDNA dynamics improved model fit for treatment response and disease progression beyond clinical factors alone. Statistical analyses were conducted by two independent statistical programmers using R statistical software (R Foundation, Vienna, Austria), version 4.3.3.

## Inclusion and ethics

One or more of the authors of this paper self-identifies as an underrepresented ethnic and/or gender minority in science. We support inclusive, diverse, and equitable conduct of research.

## Supplementary information

Supplementary Material

## Data Availability

Data collected for the study, including de-identified participant data, will be made available to others at publication via a signed data access agreement at the discretion of the investigators.

## Code availability

The code generated for the study is not publicly available. Reasonable requests will be considered at the discretion of the investigators and dependent on the proposed use of the code.
