# Supplementary material for: Clinical validation of a tissue-agnostic genome-wide methylome enrichment assay to monitor response to pembrolizumab
Source: NPJ Precis Oncol. 2026 Feb 13;10:129. doi: 10.1038/s41698-026-01327-y (PMC13013639; doi:10.1038/s41698-026-01327-y)
Supplement: Supplementary file 1 — Supplementary Material [file 41698_2026_1327_MOESM1_ESM.pdf]

Supplementary Material

Clinical validation of a tissue-agnostic genome-wide methylome enrichment assay to monitor response to pembrolizumab

Eric Y. Stutheit-Zhao[1], Yongqi Zhong[2], Collin A. Melton[2], Elizabeth D. Lightbody[2], Michael A. Hinterberg[2], Yarong Wang[2], Owen Hall[2], Eduardo V. Sosa[2], Jeremy B. Provance[2], Junjun Zhang[2], Abel Licon[2], Zhihui Amy Liu[1], Albiruni R. Abdul Razak[1], Anna Spreafico[1], Philippe L. Bedard[1], Aaron R. Hansen[1], Stephanie Lheureux[1], Pamela S. Ohashi[1,3], Alan Williams[2], Scott V. Bratman[1,4], Brian A. Allen[2], Jing Zhang[2], Daniel D. De Carvalho[1,4], Anne-Renee Hartman[2], Lillian L. Siu[1], Enrique Sanz-Garcia[1]

[1]Princess Margaret Cancer Centre, University Health Network, Toronto, Ontario, Canada
[2]Adela, Inc., Foster City, CA, USA
[3]Department of Immunology, University of Toronto, Toronto, Ontario, Canada
[4]Department of Medical Biophysics, Temerty Faculty of Medicine, University of Toronto, Toronto, Ontario, Canada

Supplementary Materials	
Supplementary Figure 1	Page 2
Supplementary Figure 2	Page 3
Supplementary Table 1	Page 4
Supplementary Table 2	Page 5
Supplementary Table 3	Page 6
Supplementary Table 4	Page 7
Supplementary Table 5	Page 8
Supplementary Table 6	Page 9
Supplementary Table 7	Page 10
Supplementary Table 8	Page 11

Supplementary Figure 1. Relative ctDNA scores in serial blood draws over the course of treatment across tumor types

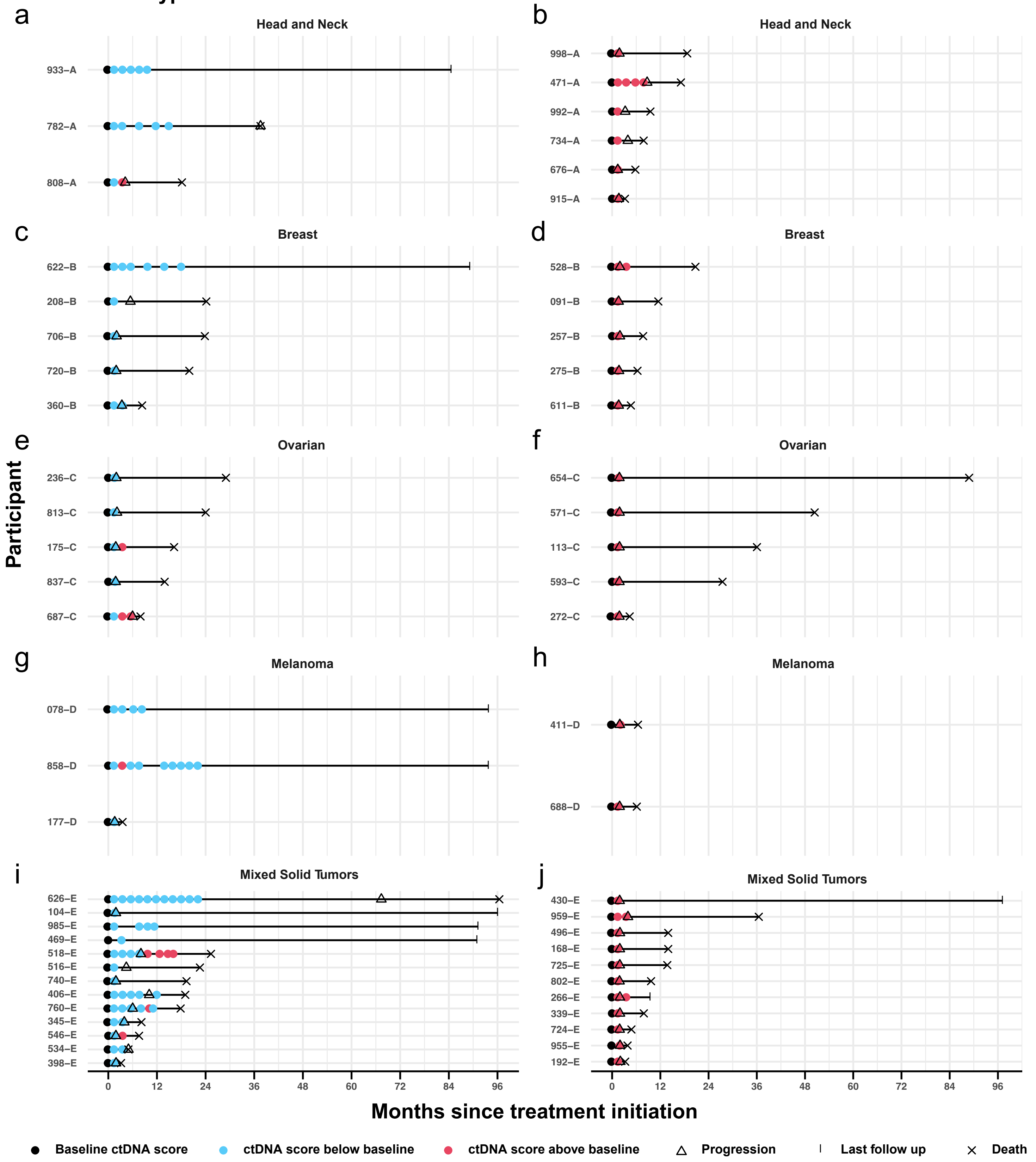

Swimmer plots show on-treatment ctDNA score relative to the baseline ctDNA score in available serial blood draws over the course of treatment and clinical outcomes for individual participants, stratified by tumor type: head and neck **(a,b)**, breast **(c,d)**, ovarian **(e,f)**, melanoma **(g,h)**, mixed solid tumors **(i,j)**. Swimmer plots depicting on-treatment ctDNA score relative to the baseline ctDNA score in available serial blood draws over the course of treatment in each patient eligible for analysis of ctDNA change from baseline to C3 (n=183 samples from 58 patients). Each row represents a unique patient participant (y-axis) where patients are grouped by ctDNA score status at cycle 3 and ordered by total length of follow-up since treatment initiation (x-axis, months). Patients with ctDNA score <baseline ctDNA score at cycle 3 (n=29) (blue circles; left panel) and patients with ctDNA score >baseline ctDNA score at cycle 3 (n=29) (pink circles; right panel) are shown. Black circles indicate baseline ctDNA samples, open triangles indicate disease progression, 'X' indicate death events, and 'ticks' represent last follow-up. ctDNA, circulating tumor DNA.

Supplementary Figure 2. Changes in ctDNA scores from baseline to cycle 3 across cancer cohorts

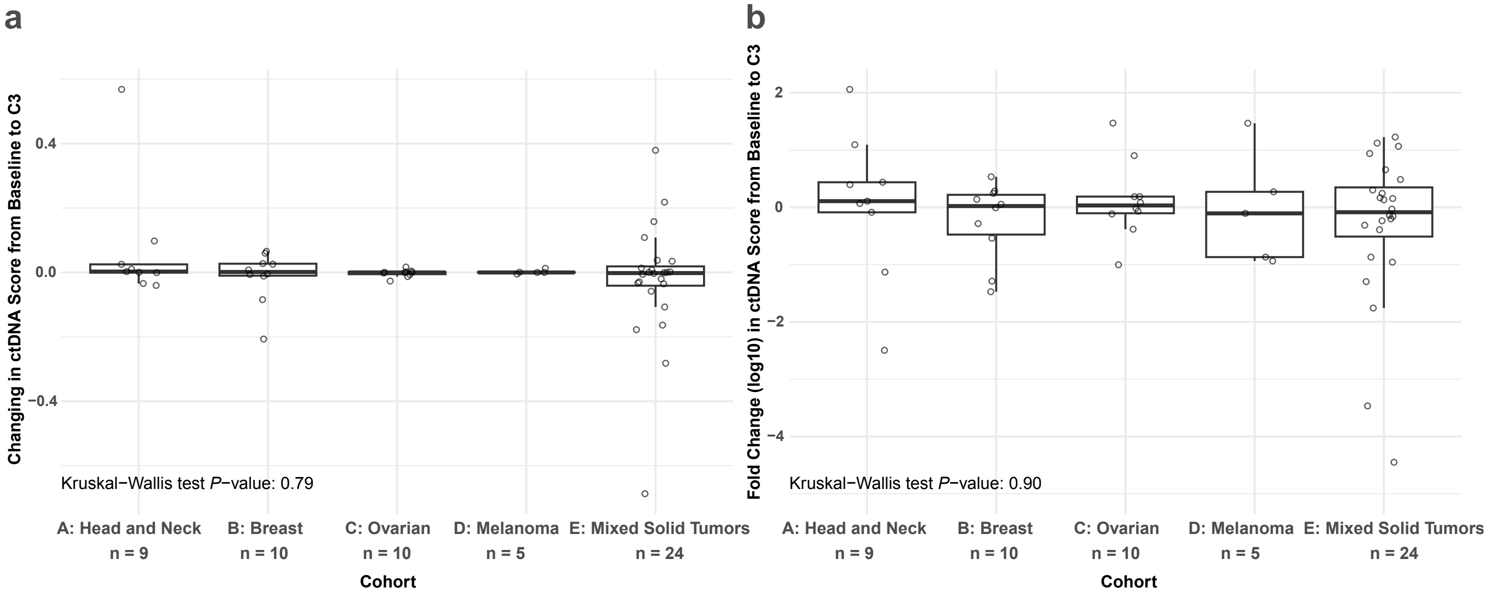

a) Absolute change in ctDNA score from baseline to cycle 3 for patients with head and neck cancer (n=9), breast cancer (n=10), ovarian cancer (n=10), melanoma (n=5), and mixed solid tumors (n=24).
b) Fold change (log10) in ctDNA score from baseline to cycle 3 across the same cancer cohorts. Boxplots display the median, interquartile range, and individual patient values. P-values were calculated using the Kruskal-Wallis test.

Supplementary Table 1. Patient demographics and clinical characteristics for included (n=58) and excluded (n=47) patients

Variable	Summary	Overall (n=95)	Excluded (n=37)	Included (n=58)	*P*-value*
**Age**	Median (Q1, Q3)	59.79 (49.68, 68.26)	61.20 (50.96, 66.19)	59.22 (49.55, 69.10)	0.82
**Sex**	Female	60 (63.16%)	27 (72.97%)	33 (56.90%)	0.13
	Male	35 (36.84%)	10 (27.03%)	25 (43.10%)	
**Cohort**	A. Head and Neck	15 (15.79%)	6 (16.22%)	9 (15.52%)	0.11 (comparison among 5 cohorts)
	HPV+	4 (26.67%)	1 (16.67%)	3 (33.33%)	0.50 (for HPV status within Cohort A)
	HPV-	4 (26.67%)	3 (50.00%)	1 (11.11%)	
	Unknown	5 (33.33%)	2 (33.33%)	3 (33.33%)	
	Missing	2 (13.33%)	0 (0.00%)	2 (22.22%)	
	B. Breast	19 (20.00%)	9 (24.32%)	10 (17.24%)	
	C. Ovarian	20 (21.05%)	10 (27.03%)	10 (17.24%)	
	D. Melanoma	11 (11.58%)	6 (16.22%)	5 (8.62%)	
	E. Mixed Solid Tumors	30 (31.58%)	6 (16.22%)	24 (41.38%)	
**Best Overall Response**	Complete Response	3 (3.16%)	2 (5.41%)	1 (1.72%)	0.10
	Partial Response	14 (14.74%)	5 (13.51%)	9 (15.52%)	
	Stable Disease	23 (24.21%)	8 (21.62%)	15 (25.86%)	
	Progressive Disease	51 (53.68%)	18 (48.65%)	33 (56.90%)	
	Not Evaluable	4 (4.21%)	4 (10.81%)	0 (0.00%)	
**PD-L1 Expression (%)**	n	92	34	58	0.74
	Mean (SD)	13.52 (29.15)	14.88 (29.89)	12.72 (28.94)	
**PD-L1 Expression**	<1%	50 (52.63%)	18 (48.65%)	32 (55.17%)	0.22
	1-49%	32 (33.68%)	12 (32.43%)	20 (34.48%)	
	≥50%	10 (10.53%)	4 (10.81%)	6 (10.34%)	
	Missing	3 (3.16%)	3 (8.11%)	0 (0.00%)	
**Tumor Mutational Burden**	n	90	34	56	0.83
	Mean (SD)	10.45 (43.82)	6.12 (11.15)	13.07 (54.90)	
**Objective Response**	Yes	17 (17.89%)	7 (18.92%)	10 (17.24%)	0.47
	No	77 (81.05%)	29 (78.38%)	48 (82.76%)	
	Not Evaluable	1 (1.05%)	1 (2.70%)	0 (0.00%)	
**Clinical Benefit**	Yes	23 (24.21%)	9 (24.32%)	14 (24.14%)	0.67
	No	71 (74.74%)	27 (72.97%)	44 (75.86%)	
	Not Evaluable	1 (1.05%)	1 (2.70%)	0 (0.00%)	
**Progression Status**	Yes	84 (88.42%)	32 (86.49%)	52 (89.66%)	0.75
	No	11 (11.58%)	5 (13.51%)	6 (10.34%)	
**Survival Status**	Deceased	80 (84.21%)	31 (83.78%)	49 (84.48%)	1.00
	Alive	15 (15.79%)	6 (16.22%)	9 (15.52%)	

Of 106 enrolled patients, 11 patients were excluded due to lack of remaining blood samples. No clinical data are available for these patients; therefore, they are not included in the overall population data.
An additional 26 patients were excluded for lack of any treatment cycle timepoints; 5 patients were excluded due to assay results being below the reportable threshold at all available timepoints.
Of the remaining 64 patients, 4 patients were excluded who had no available cycle 3 blood sample, and 2 patients were excluded who had assay results below the reportable threshold at both baseline and cycle 3 timepoints.

* Wilcoxon rank sum test with continuity correction was used for continuous variables, and Fisher's exact test was used for categorical variables.
-, negative; +, positive; HPV, human papilloma virus; PD-L1, programmed death-ligand 1; Q1/3, quartile 1/3; SD, standard deviation.

Supplementary Table 2. Proportion of patients with ctDNA increase versus decrease from baseline to cycle 3 by cancer cohort

Cohort (n)	Increase from baseline to C3 (n=29, 50%)	Decrease from baseline to C3 (n=29, 50%)
A. Head and Neck (n=9)	3 (33.33%)	6 (66.67%)
B: Breast (n=10)	5 (50.00%)	5 (50.00%)
C: Ovarian (n=10)	5 (50.00%)	5 (50.00%)
D: Melanoma (n=5)	3 (60.00%)	2 (40.00%)
E: Mixed Solid Tumors (n=24)	13 (54.17%)	11 (45.83%)

Statistical differences in the proportion of patients with increased vs decreased ctDNA from baseline to cycle 3 were assessed by Fisher's exact test (P=0.86).

C3, cycle 3.

Supplementary Table 3. Objective response univariate and multivariable analysis

Variable	Univariate analysis model Odds Ratio (95% CI)	Univariate analysis model Wald *P* value	Multivariable analysis model[1] Odds Ratio (95% CI)	Multivariable analysis model[1] Wald *P* value	Multivariable analysis model[2] Odds Ratio (95% CI)	Multivariable analysis model[2] Wald *P* value
**ctDNA score** (Reference group: Increase from baseline to C3 [n = 29])						
Decrease from baseline to C3 [n = 29*]	31.77 (3.71, 4173.19)	0.0003	33.51 (3.83, 4407.64)	0.0003	17.75 (1.79, 2365.71)	0.01
**Cohort** (Reference group: A. Head and Neck [n = 9])						
B. Breast [n = 10**]	0.47 (0.04, 4.36)	0.51	0.20 (0.01, 2.77)	0.24	0.25 (0.00, 9.91)	0.46
C. Ovarian [n = 10]	0.14 (0.00, 2.10)	0.17	0.06 (0.00, 1.15)	0.06	0.14 (0.00, 4.76)	0.27
D. Melanoma [n = 5]	2.14 (0.24, 20.69)	0.49	1.05 (0.05, 21.07)	0.97	1.61 (0.07, 46.77)	0.76
E. Mixed Solid Tumors [n = 24]	0.85 (0.16, 5.54)	0.85	0.39 (0.03, 3.67)	0.41	0.63 (0.04, 10.62)	0.72
**PD-L1 expression** [n = 58***]	1.03 (1.01, 1.05)	0.004	-	-	1.02 (1.00, 1.05)	0.13
**Tumor mutational burden** [n = 56]	1.01 (1.00, 3.13)	0.05	-	-	1.01 (1.00, 3.28)	0.17

C3, cycle 3; ctDNA, circulating tumor DNA; PD-L1, programmed death-ligand 1.
*Multivariable analysis model[2], N = 27; **multivariable analysis model[2], N = 8; ***multivariable analysis model[2], N = 56
[1]Multivariable analysis model fit using ctDNA decrease indicator and cohort.
[2]Multivariable analysis model fit using ctDNA decrease indicator, cohort, PD-L1 expression and tumor mutational burden.
Penalized likelihood ratio test comparing multivariable analysis model[2] to multivariable analysis model[2] without ctDNA decrease indicator yielded a *P*-value of 0.0009.

Supplementary Table 4. Clinical benefit univariate and multivariable analysis

Variable	Univariate analysis model Odds Ratio (95% CI)	Univariate analysis model Wald *P* value	Multivariable analysis model[1] Odds Ratio (95% CI)	Multivariable analysis model[1] Wald *P* value	Multivariable analysis model[2] Odds Ratio (95% CI)	Multivariable analysis model[2] Wald *P* value
**ctDNA score** (Reference group: Increase from baseline to C3 [n = 29])						
Decrease from baseline to C3 [n = 29*]	15.55 (3.31, 151.52)	0.0002	18.89 (3.64, 222.98)	0.0001	11.33 (1.99, 143.31)	0.004
**Cohort** (Reference group: A. Head and Neck [n = 9])						
B. Breast [n = 10**]	0.29 (0.02, 2.29)	0.25	0.11 (0.00, 1.26)	0.08	0.13 (0.00, 3.83)	0.25
C. Ovarian [n = 10]	0.29 (0.02, 2.29)	0.25	0.11 (0.00, 1.26)	0.08	0.27 (0.01, 4.66)	0.36
D. Melanoma [n = 5]	1.33 (0.15, 11.09)	0.79	0.53 (0.03, 8.08)	0.65	0.83 (0.04, 17.85)	0.90
E. Mixed Solid Tumors [n = 24]	0.80 (0.17, 4.10)	0.77	0.34 (0.03, 2.61)	0.30	0.62 (0.05, 8.08)	0.69
**PD-L1 expression** [n = 58***]	1.03 (1.01, 1.05)	0.002	-	-	1.02 (1.00, 1.05)	0.09
**Tumor mutational burden** [n = 56]	1.01 (1.00, 1.07)	0.10	-	-	1.00 (1.00, 1.08)	0.31

C3, cycle 3; ctDNA, circulating tumor DNA; PD-L1, programmed death-ligand 1.
*Multivariable analysis model[2], N = 27; **multivariable analysis model[2], N = 8; ***multivariable analysis model[2], N = 56
[1]Multivariable analysis model fit using ctDNA decrease indicator and cohort.
[2]Multivariable analysis model fit using ctDNA decrease indicator, cohort, PD-L1 expression and tumor mutational burden.
Penalized likelihood ratio test comparing multivariable analysis model[2] to multivariable analysis model[2] without ctDNA decrease indicator yielded a *P*-value of 0.002.

Supplementary Table 5. PFS univariate and multivariable analysis

Variable	Univariate analysis model<br>Hazard Ratio (95% CI)	Univariate analysis model<br>Wald *P* value	Multivariable analysis model[1]<br>Hazard Ratio (95% CI)	Multivariable analysis model[1]<br>Wald *P* value	Multivariable analysis model[2]<br>Hazard Ratio (95% CI)	Multivariable analysis model[2]<br>Wald *P* value
**ctDNA score**<br>(Reference group: Increase from baseline to C3 [n = 28])						
Decrease from baseline to C3 [n = 29*]	0.27 (0.14, 0.50)	<0.0001	0.21 (0.11, 0.43)	<0.0001	0.29 (0.15, 0.59)	0.0006
**Cohort**<br>(Reference group: A. Head and Neck [n = 9])						
B. Breast [n = 10**]	1.48 (0.57, 3.85)	0.43	2.61 (0.94, 7.24)	0.07	2.87 (0.94, 8.74)	0.06
C. Ovarian [n = 10]	2.67 (1.02, 6.99)	0.04	4.68 (1.66, 13.16)	0.003	3.60 (1.21, 10.69)	0.02
D. Melanoma [n = 4***]	0.49 (0.10, 2.32)	0.37	0.92 (0.18, 4.57)	0.92	1.01 (0.17, 5.81)	0.99
E. Mixed Solid Tumors [n = 24]	1.16 (0.52, 2.62)	0.72	1.74 (0.74, 4.07)	0.20	1.50 (0.61, 3.71)	0.38
**PD-L1 expression** [n = 57****]	0.98 (0.97, 1.00)	0.01	-	-	0.99 (0.98, 1.00)	0.07
**Tumor mutational burden** [n = 55]	0.97 (0.94, 1.01)	0.14	-	-	0.98 (0.94, 1.02)	0.33

C3, cycle 3; ctDNA, circulating tumor DNA; PD-L1, programmed death-ligand 1; PFS, progression-free survival.
*Multivariable analysis model[2], N = 27; **multivariable analysis model[2], N = 8; ***one patient excluded from PFS analysis due to progression before C3; ****multivariable analysis model[2], N = 55
[1]Multivariable analysis model fit using ctDNA decrease indicator and cohort.
[2]Multivariable analysis model fit using ctDNA decrease indicator, cohort, PD-L1 expression and tumor mutational burden.
Likelihood ratio test comparing multivariable analysis model[2] to multivariable analysis model[2] without ctDNA decrease indicator yielded a *P*-value of 0.0004.

Supplementary Table 6. OS univariate and multivariable analysis

Variable	Univariate analysis model<br>Hazard Ratio (95% CI)	Univariate analysis model<br>Wald *P* value	Multivariable analysis model[1]<br>Hazard Ratio (95% CI)	Multivariable analysis model[1]<br>Wald *P* value	Multivariable analysis model[2]<br>Hazard Ratio (95% CI)	Multivariable analysis model[2]<br>Wald *P* value
**ctDNA score**<br>(Reference group: Increase from baseline to C3 [n = 29])						
Decrease from baseline to C3 [n = 29*]	0.49 (0.27, 0.86)	0.01	0.45 (0.25, 0.83)	0.01	0.65 (0.35, 1.21)	0.17
**Cohort**<br>(Reference group: A. Head and Neck [n = 9])						
B. Breast [n = 10**]	0.98 (0.38, 2.55)	0.97	1.06 (0.41, 2.77)	0.90	0.79 (0.27, 2.25)	0.65
C. Ovarian [n = 10]	0.79 (0.31, 2.01)	0.62	0.63 (0.24, 1.66)	0.35	0.43 (0.16, 1.17)	0.10
D. Melanoma [n = 5]	0.59 (0.15, 2.25)	0.44	0.75 (0.19, 2.90)	0.67	0.73 (0.16, 3.28)	0.69
E. Mixed Solid Tumors [n = 24]	0.76 (0.33, 1.76)	0.52	0.77 (0.33, 1.79)	0.55	0.54 (0.22, 1.35)	0.19
**PD-L1 expression** [n = 58***]	0.99 (0.98, 1.00)	0.053	-	-	0.99 (0.97, 1.00)	0.046
**Tumor mutational burden** [n = 56]	0.98 (0.95, 1.02)	0.39	-	-	0.99 (0.95, 1.02)	0.46

C3, cycle 3; ctDNA, circulating tumor DNA; PD-L1, programmed death-ligand 1; OS, overall survival.
*Multivariable analysis model[2], N = 27; **multivariable analysis model[2], N = 8; ***multivariable analysis model[2], N = 56
[1]Multivariable analysis model fit using ctDNA decrease indicator and cohort.
[2]Multivariable analysis model fit using ctDNA decrease indicator, cohort, PD-L1 expression and tumor mutational burden.
Likelihood ratio test comparing multivariable analysis model[2] to multivariable analysis model[2] without ctDNA decrease indicator yielded a *P*-value of 0.17.

Supplementary Table 7. Clinical outcomes in patients with ctDNA below the reportable threshold at baseline and/or other timepoints

Reason for Exclusion	Cancer Type	Treatment Response	Progression Status	Survival Status	Absolute Change in ctDNA Score from Baseline
All ctDNA scores below reportable threshold	Melanoma	Complete Response	No	Alive	No Cycle 3 sample available and all post-baseline ctDNA scores above baseline
	Melanoma	Partial Response	No	Alive	Decrease at C3 and all post-baseline scores below baseline
	Sarcoma	Stable Disease ≥ 6 Cycles	Yes	Deceased	Decrease at C3 but at least one post-baseline score above baseline at later cycles
	Ovarian	Stable Disease < 6 Cycles	Yes	Deceased	Increase at C3
	Sarcoma	Progressive Disease	Yes	Deceased	Increase at C3
Both baseline and C3 ctDNA scores below reportable threshold	Melanoma	Partial Response	No	Alive	Increase at C3
	Adenoid Cystic Carcinoma	Stable Disease ≥ 6 Cycles	Yes	Deceased	Decrease at C3 but at least one post-baseline score above baseline at later cycles

Summary of reasons for exclusion, cancer type, treatment responses, progression and survival outcomes, and ctDNA dynamics for patients whose ctDNA remained below the reportable limit across all timepoints or at both baseline and C3.

C3, cycle 3; ctDNA, circulating tumor DNA.

Supplementary Table 8. Comparative analysis of clinical outcomes between patients with ctDNA below reportable threshold and patients included in primary analyses

Clinical Outcomes	Excluded due to scores below reportable threshold (n=7)	Included in the primary analyses (n=58)	*P*-value*
**Objective Response (CR/PR)**	42.86% (3/7)	17.24% (10/58)	0.14
**Clinical Benefit (CR/PR/SD ≥ 6 Cycles)**	71.43% (5/7)	24.14% (14/58)	*0.02*
**Progression Status (Yes)**	57.14% (4/7)	89.66% (52/58)	*0.05*
**Survival Status (Deceased)**	57.14% (4/7)	84.48% (49/58)	0.11

*Fisher's exact test evaluating the differences in clinical outcomes between patients excluded due to ctDNA scores below the reportable threshold (n=7) and those included in the primary analyses (n=58). Italicized *P*-values indicate significant differences.

CR, complete response; PR, partial response; SD, stable disease.
